# Antiviral propierties of 5,5'-dithiobis-2-nitrobenzoic acid and bacitracin against T-tropic human immunodeficiency virus type 1

**DOI:** 10.1186/1743-422X-8-137

**Published:** 2011-03-24

**Authors:** Humberto H Lara, Liliana Ixtepan-Turrent, Elsa N Garza-Treviño, Samantha M Flores-Teviño, Gadi Borkow, Cristina Rodriguez-Padilla

**Affiliations:** 1 Laboratorio de Inmunología y Virología, Departamento de Microbiología e Inmunología, Universidad Autonoma de Nuevo Leon, San Nicolas de los Garza, Nuevo Leon, Mexico; 2 Hama'ayan 4, Ligad Center 2, Modi'in 71700, Israel

## Abstract

Bacitracin and the membrane-impermeant thiol reagent 5,5'-dithiobis-2-nitrobenzoic acid (DTNB) are agents known to inhibit protein disulfide isomerase (PDI), a cell-surface protein critical in HIV-1 entry therefore they are fusion inhibitors (FI). Here we investigated the possibility that Bacitracin and or DTNB might have other antiviral activities besides FI. By means of residual activity assays, we found that both compounds showed antiviral activity only to viruses T-tropic HIV-1 strain. Cell-based fusion assays showed inhibition on HeLa-CD4-LTR-β-gal (CD4) and HL2/3 cells treated with Bacitracin, and DTNB with the latest compound we observed fusion inhibition on both cells but strikingly in HL2/3 cells (expressing Env) indicating a possible activity on both, the cell membrane and the viral envelope. A time-of-addition experiment showed that both compounds act on HIV entry inhibition but DTNB also acts at late stages of the viral cycle. Lastly, we also found evidence of long-lasting host cell protection *in vitro *by DTNB, an important pharmacodynamic parameter for a topical microbicide against virus infection, hours after the extracellular drug was removed; this protection was not rendered by Bacitracin. These drugs proved to be leading compounds for further studies against HIV showing antiviral characteristics of interest.

## Introduction

The pandemic of Human Immunodeficency Virus Tipe 1 (HIV-1) infection, the cause of AIDS, is a public health issue and ranks among the greatest infectious disease scourges in history [1]. There were more than 33.3 million people worldwide with HIV-1 infection or AIDS, according to estimates by the Joint United Nations Programme on HIV/AIDS 2009 (UNAIDS).

The use of highly active antiretroviral therapy has dramatically reduced morbidity and mortality among patients infected with HIV-1 [2,3]. However, the success of antiretroviral treatment is frequently limited by the emergence of HIV-1 drug resistance. Several researchers have attempted to develop various virucidal agents to inactivate the cell-free virions and therefore prevent the sexual transmission of HIV-1 [4-7].

In the early event of HIV-1 infection, the viral glycoprotein gp120 attaches the virus to the cell by binding to its receptor CD4 on cells of the host's immune system. Another cell-surface protein was found to be involved in HIV-1 entry, the oxidoreductase protein disulfide isomerase (PDI) [8,9]. PDI has been described to play a role in host cell uptake of some toxins, bacteria, parasite [10] and virus modulating the exchange of protons between a cell-surface associated donor (PDI) and viral proteins by catalyzes thiol-disulfide SH/-S-S- interchange [11,12].

Besides the observation that PDI inhibition blocks the HIV replicative cycle [13] by conformational changes, gp120 interacts with the enzyme PDI and the chemokine co-receptors form a PDI-CD4-gp120-chemokine complex also prevent HIV envelope mediated fusion [9] PDI can reach the complex and reduce disulfide bonds in gp120 [14] that involves cleavage of two of the nine disulfide bonds of gp120 [15-17], which causes key conformational changes in gp120 and activate gp41 enhancing the fusogenic potential of the viral envelope. It has been shown that inhibition of HIV-1 entry can be brought about by introducing membrane impermeant sulfhydryl agents that can block the redox function of PDI, acting inhibiting PDI, to prevent the viral entry [8,18].

Studies reported that the thiol reagent 5,5'-dithiobis-2-nitrobenzoic acid (DTNB) [8,9], and Bacitracin were commonly used as inhibitors of PDI activity. These agents will stop the generation of two free thiols in a gp120 and an oxidized form of CXXC motif in PDI, blocking HIV-1 infection [19]. More recently, were showed that these inhibitors altered the HIV receptor-dependent gp41-mediated fusion process *per se *at a post-CD4 binding step, particularly DTNB, prevents gp41 from assuming the 6-helix bundle conformation that drives fusion [15-17], but the effect was temporally, only was observed when the agents were present at the time of virus-cell interaction [8,9]. It is speculated that inhibition if HIV infectivity by Bacitracin could arise because of one of two effects [20]. First, Bacitracin could inhibit PDI by competing with substrate binding, especially by competing for the substrate-binding site on the b' domain. Second, PDI activity could be inhibited by the metal ions that bacitracin is known to bind. These metal ions could be coordinated by the active site cysteines of the catalytic domains of PDI, decreasing their activity.

It was reported that the DTNB causes 100% inhibition of soluble PDI activity at 1.0 mM concentration [8,9]. The mechanisms is not yet been completely elucidated however, it has been reported the DTNB acting two conformations, there were, three, two hydrogen bonds formed; thiol group of Cys37 and backbone-oxygen atom of Phe80 in PDI enzyme interacts with the two different atom of sulfur (S2 and S1) in DTNB and side chain nitrogen atom of Arg101 was also involved in the hydrogen bond interaction with O3 atom of DTNB [19]

Previous studies described molecular docking simulation to investigate the binding mechanisms of action for Bacitracin and DTNB or fusion inhibition by reorganization of the conformation of Env, however the action as antiviral and the importance of HIV receptors (CXCR4 on CD4+ lymphocytes) has not been clearly established. For that reason and searching for other antiviral activities that these leading compounds might have, we also tested if these compounds have long lasting protection against viral infection on uninfected host cells even hours after the compounds were removed form the media which is an important pharmacodynamic parameter investigated during the development of a topical microbicide agent. In addition we evaluated the stages of the viral cycle to elucidate the target sites of these compounds.

## Methods

### Reagents, cells and HIV-1 isolates

The following reagents were obtained through AIDS Research and Reference Reagent Program, NIH: HeLa-CD4-LTR-β-gal (CD4) cells from Dr. Michael Emerman; HL2/3 (Env) cells from Dr. Barbara K. Felber and Dr. George N. Pavlakis; HIV-1_IIIB_, HIV-1_BaL _and Fusion Inhibitor T-20, Integrase Inhibitor 118-D-24 and Protease Inhibitor Amprenavir from Dr. Suzanne Gartner, Dr. Mikulas Popovic and Dr. Robert Gallo. DTNB and Bacitracin were obtained from Sigma-Aldrich. UC781, a NNRTI, was kindly donated by Dr. Gadi Borkow.

### Cytotoxicity assays

A stock solution of DTNB and Bacitracin was two-fold diluted to desired concentrations in growth medium and subsequently added into wells containing 5 × 10^4 ^HeLa-CD4-LTR-β-gal cells. Microtiter plates were incubated at 37°C in a 5% CO_2 _air humidified atmosphere for 24 hours. Assessments of cell viability were carried out using a CellTiter-Glo^® ^Luminescent Cell Viability Assay (Promega). Cytotoxicity was evaluated based on the percentage cell survival relative to the infection obtained in the absence of any compound.

### Virucidal activity assays

Serial two-fold dilutions of DTNB, Bacitracin or just media as control were added to HIV-1_IIIB _or HIV-1_BaL _cell-free virus. After incubation for 5 min at room temperature, the mixtures of compounds with virus were centrifuged three times at 10,000 rpm, the supernatant fluids removed, and the pellets washed three times. The final pellets were resuspended in DMEM and placed into 96-well plates with HeLa-CD4-LTR-β-gal cells. The cells were incubated in a 5% CO_2 _humidified incubator at 37°C for 24 h. Assessment of HIV-1 infection was performed with the Beta-Glo Assay System. The percentage of residual infectivity after DTNB, Bacitracin or media as control was calculated with respect to the control. The 50% inhibitory concentration (IC_50_) was defined according to the percentage of infectivity inhibition relative to the positive control.

### Cell-based fusion assay

HeLa-derived HL2/3 cells, which express the HIV-1_HXB2 _Env, Tat, Gag, Rev, and Nef proteins, were co-cultured with HeLa-CD4-LTR-β-gal cells at a 1:1 cell density ratio (5 × 10^4 ^cells/well each) for 24 h in the absence or presence of two-fold dilutions of DTNB, Bacitracin, UC781, and T-20 in order to examine whether the compounds interfered with the binding process of HIV-1 Env and the CD4 receptor. Also, both HeLa-CD4-LTR-β-gal and HL2/3 cells were exposed independently only 30 minutes to the aforementioned compounds and then washed twice thoroughly to eliminate residual compound before co-cultivating with the other cell line. Upon fusion of both cell lines, the Tat protein from HL2/3 cells activates β-galactosidase indicator gene expression in HeLa-CD4-LTR-β-gal cells ^20^. β-gal activity was quantified with the Beta-Glo Assay System (Promega). The percentage of inhibition of HL2/3-HeLa CD4 cell fusion was calculated with respect to the positive control of untreated cells.

### Time-of-addition experiments

HeLa-CD4-LTR-β-gal cells were infected with 10^5 ^TCID_50 _of HIV-1_IIIB _cell-free virus with a 0.2-0.5 MOI. Bacitracin (3.5 mM), DTNB (6 mM), T-20 (100 μM), UC781 (70 nM), 118-D-24 (120 μM) and Amprenavir (0.1 mM) were then added upon HIV-1 inoculation (time zero) or at various time points post-inoculation. The reference compounds were added at a concentration several times their EC_50 _required to reduce by 50% the cytopathicity of HIV-1_IIIB_. Infection inhibition was quantified after 24 h of incubation, by measuring β-gal activity of the target cells with the Beta-Glo Assay System.

### Cell-Protection Assays

HeLa-CD4-LTR-β-gal cells were incubated with DTNB (12.6 mM) or Bacitracin (5.3 mM) for 30 minutes and subsequently the cells were washed with PBS three times. Then, the cells were infected with 10^5 ^TCID_50 _of HIV-1_IIIB _cell-free virus with a 0.2-0.5 MOI at different times (1, 3, 6, 10 and 24 h) of exposure to the drugs. The inhibition of infection was quantified after 24 h by measuring β-gal activity with the Beta-Glo Assay System.

### Statistical analysis

Graphs were done with SigmaPlot 10.0 software and the values shown are means ± standard deviations from three separate experiments, each of which was carried out in duplicate.

## Results

### Range of virucidal activity

**Figure 1:**
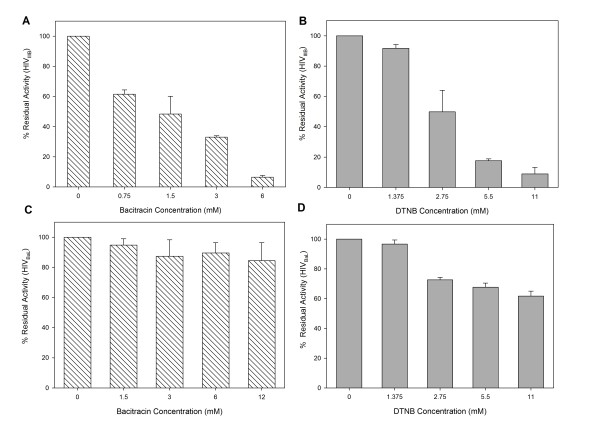
**Residual Activity on HIV-1 strains**. **A, B) **HIV-1_IIIB _and **C, D) **HIV-1_BaL _cell-free viruses were exposed to serial dilutions of **A, C) **Bacitracin and **B, D) **DTNB for 5 minutes. The viruses were then ultracentrifuged, washed three times and added to HeLa-CD4-LTR-β-gal cells After 24 hours, β-gal activity was measured. Percentage values are relative to the positive control (no compound pretreatment).

To study the effect that DTNB or Bacitracin have over the virus itself, M-tropic (HIV-1_BaL_) and T-tropic (HIV-1_IIIB_) isolates were treated with different concentrations of PDI inhibitors the mixtures were centrifuged three times at 10,000 rpm in an eppendrof centrifuge. After removal of the supernatant fluids with or without (control) compounds, the pellets were washed three times. The final pellets were placed into 96-well plates with HeLa-CD4-LTR-β-gal cells. Assessment of HIV-1 infection was made with a luciferase-based assay. The percentage of residual infectivity after treatment was calculated with respect to the positive control of untreated virus. The assay was performed in triplicate; the data points represent the mean, and the solid lines are nonlinear regression curves done with SigmaPlot 10.0 software.by centrifugation, the residual infectivity of the cell-free viruses was quantified by a luciferase-based assay using indicator cell lines. As shown in Figure 1(A-B), pretreatment with Bacitracin or DTNB reduced the infectivity of T-tropic (HIV-1_IIIB_) after just 5 min of exposure. However, both compounds pretreatment did not show a significant inhibition activity over M-tropic (HIV-1_BaL_). The concentration of Bacitracin and DTNB at which HIV-1_IIIB _infectivity was inhibited by 50% (IC_50_) was 1.18 mM and 2.60 mM, respectively, Bacitracin showed a higher therapeutic index (TI), more than three times higher than the TI showed by DTNB. However, cytotoxic concentration which 50% of cell dead (CC_50_) were 30.37 and 19.77 for Bacitracin and DTNB respectly (Table 1).

**Table 1:** CC_50 _and virucidal IC_50 _values for HIV-1 strains.

	CC_50 _(mM)	IC_50 _(mM)		TI (HIV-1_IIIB_/HIV-1_Ba-L_)
			
		HIV-1_IIIB_	HIV-1_Ba-L_	
**Bacitracin**	30.37	1.18	> 30.37	25.73/< 1

**DTNB**	19.77	2.60	> 19.77	7.60/< 1

### Inhibition of Env/CD4-mediated membrane fusion

**Figure 2:**
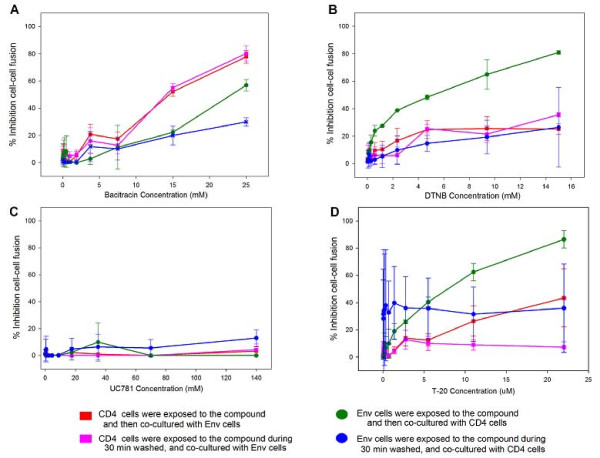
**Inhibition of Env/CD4-mediated membrane fusion**. β-gal activity was measured after CD4 and Env cells were co-cultured when exposed to **A) **Bacitracin, **B) **DNTB, **C) **UC781 and **D) **T-20, in different circumstances: (red) CD4 cells were exposed to the compound and co-cultured with Env cells for 24 hours. (green) Env cells were exposed to the compound and co-cultured with CD4 cells for 24 hours. (purple) CD4 cells were exposed to the compound for 30 minutes, washed, and co-cultured with Env cells for 24 hours. (blue) Env cells were exposed to the compound for 30 minutes, washed, and co-cultured with CD4 cells for 24 hours. Percentage values are relative to the positive control (no treatment). The data represent the means ± standard deviations from three separate experiments, each of which was carried out in duplicate.

A cell-based fusion assay was used to mimic the gp120-CD4-mediated fusion process between HIV-1 and the host cell. The effector cells were HL2/3 cells, which express HIV-1 Env on their surfaces and Tat protein in their cytoplasms [21], and experiments were performed to determinate whether DTNB and Bacitracin tended to act directly either on the HeLa-CD4-LTR-β-gal (CD4) cells or HL2/3 (Env) cells or boths cells when inhibiting fusogenic activity. When HeLa-CD4-LTR-β-gal (CD4) cells were first exposed to the compounds and then HL2/3 (Env) cells were added, both Bacitracin (Figure 2A) and DTNB (Figure 2B) blocked fusion in a dose response manner between both cells; the same happened when HL2/3 (Env) cells were first exposed to the compounds and then HeLa-CD4-LTR-β-gal (CD4) cells were added (Figure 2A-B). A significant lower dose of Bacitracin was required to reach an observable decrease in fusogenic activity in HeLa-CD4-LTR-β-gal (CD4) cells than the dose needed to pretreat HL2/3 (Env) cells (p = 0.09); on the other hand, a significant lower dose of DTNB was needed to inhibit the fusogenic activity when HL2/3 (Env) cells were pretreated compared to the pretreatment of HeLa-CD4-LTR-β-gal (CD4) cells (p = 0.009). In addition the assays were performed, while the exposure of Bacitracin and DTNB to HeLa-CD4-LTR-β-gal (CD4) or HL2/3 (Env) cells, separately, during 30 minutes only, following by extracellular compound removal by washing the cells. Results showed a significant lower dose was required to inhibit fusogenic activity when HL2/3 (Env) cells were pretreated with DTNB during 24 hours than the 30 minutes pretreatment before mixing to HeLa-CD4-LTR-β-gal (CD4) cells exposure (p = 0.007). Our data, as well as data from others laboratories, support the concept that thiol/disulfide rearrangement in gp120 catalyzed by PDI occurs post-CD4 engagement [17]. Known antiretroviral drugs were used as controls, such as UC781 (NNRTI), which did not inhibit cell fusion in these cell-based fusion assays (Figure 2C), and T-20 (FI), which did inhibit cell fusion in all assays, except when exposed to HeLa-CD4-LTR-β-gal (CD4) cells for only 30 minutes (Figure 2D).

### Time (Site) of Intervention

**Figure 3:**
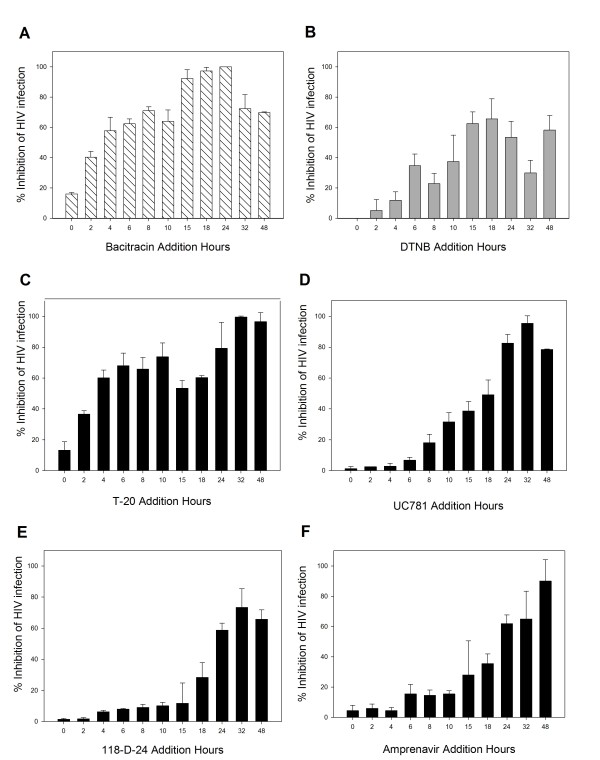
**Time of Intervention in HIV-1 Life Cycle**. HeLa/CD4-LTR-β-gal cells were infected with HIV-1_IIIB _cell-free virus before **A**) Bacitracin (3.5 mM), **B**) DTNB (6 mM), **C**) T-20 (100 μM), **D**) UC781 (70 nM), **E**) 118-D-24 (120 μM) and **F**) Amprenavir (0.1 mM), were added upon HIV-1 inoculation (time zero) or at various time points post-inoculation and β-gal activity was measured following 24 hr of incubation. Percentage values are relative to the positive control (no treatment). The data represent the means ± standard deviations from three separate experiments, each of which was carried out in duplicate.

To further determine the antiviral target of Bacitracin and DTNB, a time-of-addition experiment was performed using a single cycle infection assay. Several antiretroviral drugs were chosen as controls as they point out different stages of the viral cycle (fusion or entry, retrotranscription, protease activity, and integration to the genome). As seen in Figure 3(A-F), the antiviral activity of T-20, UC781, 118-D-24 and Amprenavir started to decline after the cycle stage that the antiviral target has passed. The fusion inhibitor's activity declined after 2 h (Figure 3C), RT inhibitor after 15 h (Figure 3D), integrase inhibitor after 18 h (Figure 3E), and protease inhibitor after 18 h (Figure 3F). Bacitracin retained its antiviral activities during 2 h (Figure 3A) as a fusion inhibitor after the HIV inoculation. On the other hand, 15 hours after HIV inoculation, the antiviral activity of DTNB started to decrease (Figure 3B), which is the time period of viral entry and integration stages. Also, the antiviral activity of DTNB regained strength at 24 hours post-HIV inoculation, which corresponds to viral protease stage [22].

### Cell-Protection Assays

**Figure 4:**
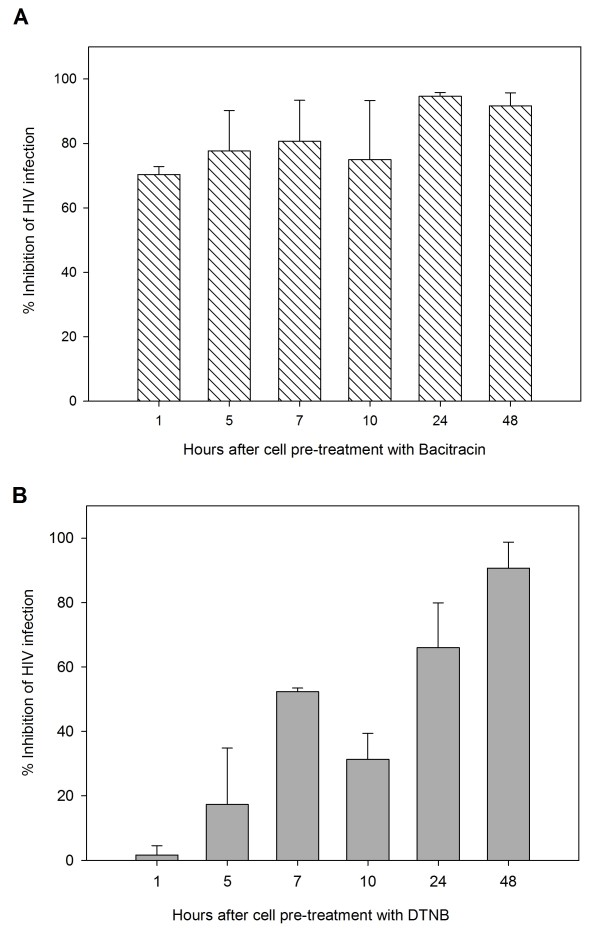
**Cell Protection Against HIV-1 Infection**. β-gal activity was measured after HeLa/CD4-LTR-β-gal cells were exposed to **A**) Bacitracin (5.3 mM) and **B**) DTNB (12.6 mM) for 30 minutes, washed and exposed to HIV-1_IIIB _cell-free virus at various time points post-treatment. Percentage values are relative to the positive control (no treatment). The data represent the means ± standard deviations from three separate experiments, each of which was carried out in duplicate.

HeLa cells were pretreated with Bacitracin and DTNB for 30 minutes and subsequently exposed to HIV-1_IIIB _at different post-infection times until 48 hours to measure cell protection without extracellular drug. As shown in Figure 4(A), after extracellular Bacitracin is removed, there is no cellular protection against infectivity, while DTNB protection against infection to cells after removal of extracellular drug, was retained until 10 hours.

## Discussion

PDI inhibition blocks the HIV early replicative cycle [7,13] and HIV entry occur at 3 different steps: CD4 binding, the gp41 mediated membrane fusion process, and during uncoating events. Thus conformational changes, gp120 interacts with the enzyme PDI and the chemokine co-receptors form a PDI-CD4-gp120-chemokine complex also prevent HIV envelope mediated fusion to the host cell [8,9,14,17,23].

DTNB and Bacitracin are PDI inhibitors that previously reported activity against HIV-1 T-tropic strains, which have CXCR4 co-receptor dependence (CXCR4) [8,9]. DTNB is a sulfhydryl blocker that covalently reacts with the catalytic sites of PDI and abolishes its capacity for cleaving disulfides, whereas Bacitracin inhibits both its reductive and oxidative functions [14,24]. In this paper we found that both compounds showed antiviral and virucidal activities in a dose-dependent manner. However T-tropic (HIV_IIIB_) strains which has CXCR4 co-receptor were susceptible to both compounds and not to the M-tropic (HIV-_BaL_) strain (Figure 1A-D) due probably at the increased of negative charge (T-tropic) in variable region (V3) of human immunodeficiency virus type 1 (HIV-1) envelope gp120 subunit participates in determination of viral infection coreceptor tropism [25-27].

Furthermore, cell-based fusion assays [28] were performed for these compounds, using HeLa-CD4-LTR-β-gal cells [29] and H2/3 (Env-expressing) cells [21], where co-culture expressing cells results in efficient cell fusion within 6-12 hours. We found that both Bacitracin and DTNB showed inhibition of cell-cell fusion in a dose-dependent manner when exposed to HeLa-CD4-LTR-β-gal cells (Figure 2A-B). These results are similar to those presented by Fenouillet *et al *[14], where PDI inhibitors as well as anti-PDI antibodies, were used to inhibit cell-cell fusion assays. However exposure to HeLa-CD4-LTR-β-gal cells showed no significant difference on cell-cell fusion on either the 24 hour or the 30 minute exposure assay which is the time the process of conformational changes of gp120 after CD4 bonding takes place [30]. Alternatively, long-term exposure (24 hours) of DTNB on HL2/3 (Env-expressing) cells and then mixing them with CD4 cells showed significant inhibition of cell-cell fusion as showed in Figure 2B. This effect of Bacitracin and DTNB markedly on Env expressing cells has never been reported.

Also, our findings indicate that Bacitracin and DTNB act differently on HeLa-CD4-LTR-β-gal cells and HL2/3 (Env-expressing) cells, given that Bacitracin has more effect on HeLa-CD4-LTR-β-gal cells but less effect against HL2/3 (Env-expressing) cells fusion's activity, however DTNB has cell-cell fusion inhibition effect on HeLa-CD4-LTR-β-gal cells but predominantly on HL2/3 (Env-expressing) cells. This data confirmed that both compounds act on the HIV receptor dependent gp41 mediated fusion process per se through inhibition of a post-CD4 binding step that has antiviral effects and virucidal action [14]. However our results with Bacitracin showed that has a better inhibition effect on the host CD4 cell acting only as fusion inhibitor on CD4 cells [20] but also having an inhibition effect on env expressing cells.

In addition, to further determine the antiviral target of Bacitracin and DTNB, a time-of addition experiment was used to delimit the stage(s) of the viral life cycle that is blocked by these compounds separately, comparing it with several antiretroviral drugs as controls that point out different stages of the viral cycle [31-36] (Figure 3C-F). Our findings suggest that Bacitracin inhibits HIV-1 infection at stage of viral entry or fusion (Figure 3A), however DTNB acts at a time period between and including viral entry and other later stages (Figure 3B). DTNB results shows that has probably an antiviral function affect on protease activity, this results are also according to Lebon [22]. This is the first time, to our knowledge, that the activity of this compound has been associated to HIV-1 life cycle stages besides fusion. However studies has shown that down regulation of PDI using small interfering RNA had only a small effect on infection or cell fusion mediated by HIV-1, suggesting that other thiol active enzymes at the cell surfaces are involved in reduction of the HIV envelope glycoprotein, that is therefore an interesting result.

Lastly, in order to provide a barrier to infection by residual active virus on uninfected cells, we evaluated the long-term effectiveness of the compound, which is an important pharmacodynamic parameter tested for the development of a topical agent [37], and is defined as the length of time that infection is suppressed following brief exposure to the antiviral agent. Ideally, a microbicide should remain effective for several hours after topical application. These compounds should be able to give cell protection for several hours after the removal of the extracellular drug [38] Although Bacitracin did not show cell protection (Figure 4A), DTNB on the other hand, showed lasting cell protection (50% infection inhibition) even 10 hours after compound was washed and removed from the indicator cells (Figure 4B). These results are consistent with a time-of addition experiment that showed that DTNB act after viral entry suggesting that DTNB somehow bonds to target cell strongly and though DTNB's PDI bonding activity is not discarded, other cell sites could be participating. Previous reports showed DTNB only has effect when anti-PDI was present at the time of virus-cell interaction (Ryser et al., 1994). Our results demonstrated that exposure of uninfected cells to DTNB renders these cells refractory to subsequent HIV-1 infection, even in the absence of a continued extracellular presence of the drug. Thus, DTNB may serve to minimize the sexual transmission of HIV-1 from infected to noninfected individuals.

In summary, although both, Bacitracin and DTNB, are classified as fusion inhibitors of cell PDI, our findings suggest new data. The data presented here are novel in that they prove that both Bacitracin and DTNB (besides acting on cell PDI), are also virucidal agents against T-tropic HIV-1 infection, and DTNB acts not only at early viral cycle stages but also at late stages with long lasting effects on the CD4 cells.

Based on our results and the above requirements, DTNB could be considered as a leading compound to further studies to determine their potential use as therapeutic agents in HIV-1 infection, especially due to their virucidal and fusion inhibitor properties.

## Competing interests

The authors declare that they have no competing interests.

## Authors' contributions

All authors read and approved the final manuscript. HHL participated in the conception and experimental design of the *in vitro *HIV-1 manipulation and infectivity assays, in analysis and interpretation of the data, and in writing and revision of this report. ENG participated in the conception and design of the *in vitro *HIV-1 manipulation and infectivity assays, in analysis and interpretation of the data, and in writing and revision of this report. LIT participated in collection of *in vitro *HIV-1 manipulation and infectivity assays. GB and CR-P. participated in the experimental design of this research.
